# HIF-1α as a Central Regulator of Monocyte Responses to Hypoxia

**DOI:** 10.3390/biology15030213

**Published:** 2026-01-23

**Authors:** Nadia Lampiasi, Roberta Russo

**Affiliations:** Istituto per la Ricerca e l’Innovazione Biomedica (IRIB), Consiglio Nazionale delle Ricerche, Via Ugo La Malfa 153, 90146 Palermo, Italy; roberta.russo@irib.cnr.it

**Keywords:** innate immunity, inflammation, cytokines, NF-κB, HIF-1α, macrophages

## Abstract

Hypoxia is a common feature of inflamed and ischemic tissues and represents an important regulatory signal for innate immune cells. The master regulator of this response is hypoxia-inducible factor-1α (HIF-1α), a transcription factor whose stabilization and activity are tightly regulated by the presence of oxygen, inflammatory signaling, and cellular metabolism. Monocytes, key players in innate immunity, rapidly sense oxygen deprivation and display specific responses during acute hypoxia, primarily aimed at adapting and maintaining cellular homeostasis. Unlike macrophages, in which HIF-1α activity is known, the mechanisms regulating HIF-1α stabilization, subcellular localization, and transcriptional activity in circulating monocytes remain incompletely elucidated. Recent studies indicate that acute hypoxia primarily triggers post-translational stabilization of HIF-1α, calcium- and PKC-dependent signaling, metabolic reprogramming, and early inflammatory responses, while transcriptional activation of HIF-1α may require additional inflammatory or stress-related signals. Furthermore, extensive crosstalk between HIF-1α and NF-κB integrates hypoxic and inflammatory signals, modulating cytokine production, cell migration, and survival. Epigenetic regulators can also modulate these responses and contribute to hypoxia-induced trained immunity. In this review, we summarize current knowledge of the mechanisms controlling the stabilization, localization, and function of HIF-1α in human monocytes and monocyte–macrophages during acute hypoxia, highlighting the key differences between these cell types and discussing their implications for inflammation, tissue homeostasis, and disease.

## Simple Summary

Hypoxia is a condition in which cells and tissues experience a decrease in molecular oxygen levels. Hypoxia causes cells to reorganize metabolically and transcriptionally to adapt to the new stressful situation. Blood monocytes are sentinel cells of our immune system that respond early to hypoxia. A key regulator of this adaptation is hypoxia-inducible factor-1α (HIF-1α), a protein that controls genes involved in metabolism, inflammation, and cell survival. The strategies monocytes adopt allow for the restoration of homeostasis as soon as the hypoxic condition ends. Conversely, if hypoxia persists for a long time or if monocytes fail to adapt, a pathological condition can develop.

## 1. Introduction

Hypoxia is the reduced availability of molecular oxygen (O_2_) to cells and tissues, a condition that leads to metabolic and oxidative stress. Hypoxia plays a fundamental role in some pathological processes, including inflammation, ischemia, and tumor progression, but tissues can also be hypoxic in some non-pathological conditions. The ability of cells to sense and adapt to reduced oxygen levels is present in all cells through an oxygen tension-sensitive transcription factor called hypoxia-inducible factor (HIF). This transcription factor is a heterodimeric complex consisting of an unstable oxygen tension-sensitive α subunit (HIF-1α) and a stable β subunit, constitutively expressed in cells (HIF-1β/ARNT). The stability of HIF-1α in the cell cytoplasm is controlled by two distinct mechanisms: (1) proteasomal degradation following the hydroxylation of two highly conserved prolyl residues (Pro-402 and Pro-564), by prolyl hydroxylase domain (PHD) family enzymes [1]; and (2) transcriptional inactivation of HIF-1α by factor-inhibiting HIF (FIH), which dissociates the p300/CBP coactivator from HIF-1α via the oxygen tension-dependent hydroxylation of asparaginyl residues [2].

**Figure 1:**
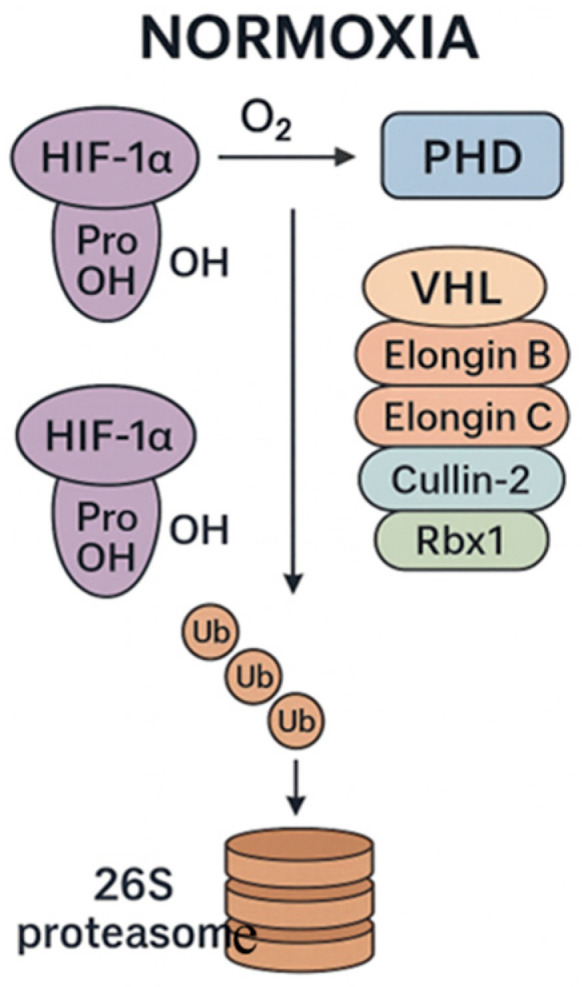
Main events leading to HIF-1 degradation under normoxia. prolyl hydroxylase domain (PHD); von Hippel–Lindau tumor suppressor protein (VHL); Elongin B, Elongin C, Cullin-2, and Rbx1 form the E3 ubiquitin ligase complex that ubiquitinates HIF-1α and targets it for degradation by the proteasome.

Under physiological oxygen conditions (normoxia), HIF-1α is synthesized but rapidly degraded by the proteasome through the activity of PHD enzymes and recognition by an E3 ubiquitin ligase complex (formed by von Hippel–Lindau tumor suppressor protein (VHL), Elongin B, Elongin C, Cullin-2, and Rbx1). Consequently, HIF-1α is polyubiquitinated and targeted for proteasomal degradation, thus inhibiting its accumulation and transcriptional activity (Figure 1) [3].

**Figure 2:**
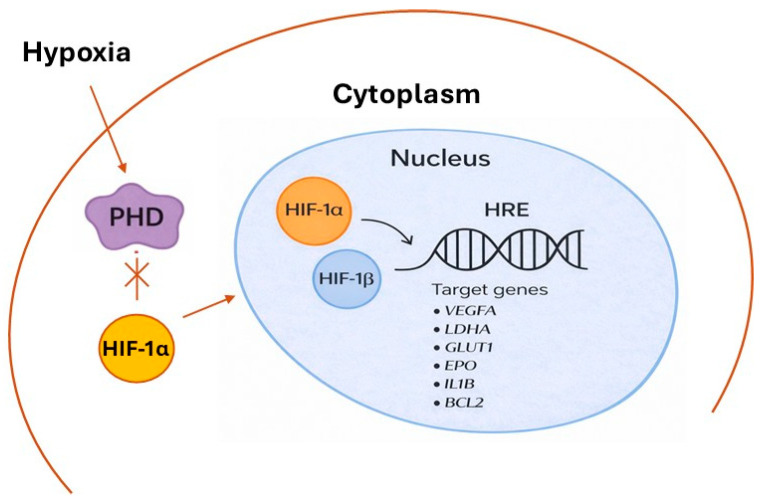
Main events leading to HIF-1 stabilization/activation under hypoxia. Oxygen starvation inhibits PHD activity, preventing the hydroxylation and subsequent degradation of HIF-1α. The stabilized protein accumulates in the cytoplasm and moves to the nucleus, where it heterodimerizes with HIF-1β and binds to HREs in DNA. This complex activates the transcription of target genes: vascular endothelial growth factor A (*VEGFA*), Lactate Dehydrogenase A (*LDHA*), glucose transporter 1 (*GLUT1*), erythropoietin (*EPO*), Interleukin-1 β (*IL1B*), and B-Cell Lymphoma 2 (*BCL2*).

On the contrary, under hypoxic conditions, cells activate adaptive transcriptional programs that depend on HIF-1α activation. In this case, the activity of PHD enzymes is inhibited due to the limited availability of molecular oxygen, which serves as an essential co-substrate for hydroxylation. Consequently, HIF-1α remains non-hydroxylated and non-ubiquitinated, and does not undergo degradation by the proteasome. Stabilized HIF-1α accumulates in the cytoplasm, and in turn moves to the nucleus, where it forms a heterodimer with HIF-1β, generating the active transcriptional complex. This complex binds to hypoxia response elements (HREs) in the promoters of several target genes involved in processes such as angiogenesis (*VEGFA*), glycolytic metabolism (*LDHA*, *GLUT1*), erythropoiesis (*EPO*), inflammation (*IL1Β*), cell survival (*BCL2*), and migration (*CXCR4*) (Figure 2) [4,5]. Through this transcriptional reprogramming, HIF-1α allows cells and tissues to adapt to oxygen starvation and plays a critical role in maintaining homeostasis under hypoxic stress.

Monocytes are essential components of the innate immune system, originating from hematopoietic stem progenitor cells (HSPCs) in the bone marrow and circulating in the bloodstream before migrating into tissues, where they differentiate into macrophages or dendritic cells (DCs) [6]. They play a role in tissue homeostasis, host defense, inflammation, and tissue remodeling [7]. Importantly, monocytes represent a heterogeneous population, with subsets characterized by differential expressions of the surface markers CD14 and CD16. Classical monocytes (CD14^+^CD16^−^) exhibit predominantly pro-inflammatory properties, non-classical monocytes (CD14^++^CD16^low^) contribute to vascular homeostasis and tissue repair, and intermediate monocytes (CD14^++^CD16^+^) most likely represent a transition state between the classical and the non-classical monocyte phenotypes [8]. Given their plasticity, migratory capacity, and central role at the interface between innate and adaptive immunity, alterations in the distribution and activation of monocyte subsets are closely associated with various pathological conditions [8,9].

In inflamed or hypoxic tissues, monocytes are recruited and induced to differentiate into macrophages, which adopt gene expression programs that allow for adaptation to low oxygen availability [10]. Macrophages are also highly plastic cells, capable of adapting to multiple situations by producing pro- and anti-inflammatory cytokines and chemokines, with the primary role of maintaining tissue homeostasis and host defense. Macrophages comprise a plethora of phenotypes, of which the pro-inflammatory M1-like and anti-inflammatory M2-like phenotypes are the functional extremes [11]. Interestingly, both monocytes and macrophages are implicated in acute hypoxia with strategies for an early and rapid response aimed at maintaining cell viability, avoiding damage and restoring homeostasis.

Because monocytes can stabilize HIF-1α in response to hypoxia, understanding how monocytes sense and adapt to hypoxic environments is of paramount importance. In this review, we summarize current knowledge of the mechanisms regulating the stabilization and subcellular localization of HIF-1α in human monocytes and monocyte–macrophages under acute hypoxic conditions. We also explore the downstream transcriptional and metabolic programs activated by HIF-1α, the crosstalk with NF-ĸB, and epigenetic regulation, and highlight the key differences between monocytes and monocyte–macrophages in regulatory pathways and functional outcomes.

## 2. Monocytes and Acute Hypoxia: Mechanisms and Functional Implications

Acute hypoxia occurs within minutes and triggers immediate and highly dynamic responses in circulating and tissue-infiltrating monocytes. Unlike chronic hypoxia, which promotes long-term transcriptional and epigenetic adaptation, acute oxygen deprivation primarily triggers post-translational stabilization of HIF-1α and metabolic reprogramming events that rapidly influence inflammatory signaling, cytoskeletal dynamics, and cellular trafficking [12]. These early adaptations allow monocytes to preserve homeostasis and maintain functions during transient episodes of oxygen limitation, such as those occurring in ischemic or inflamed tissues, and at high altitude.

### 2.1. Stabilization of HIF-1α

HIF-1α critically regulates hypoxia-driven reprogramming of monocyte and macrophage metabolism and effector functions, including glycolysis, cytokine production, migration, and survival [10]. However, the precise molecular mechanisms by which HIF-1α orchestrates monocyte adaptation to hypoxia and influences monocyte–macrophage differentiation remain incompletely understood. Many studies demonstrate that stabilization of HIF-1α in the cytoplasm of monocytes does not result in its translocation to the nucleus. For instance, primary human CD14^+^ monocytes, although able to stabilize HIF-1α under hypoxic conditions, retain it predominantly in the cytoplasm unless they are stimulated with inflammatory-like signals (lipopolysaccharide (LPS)) [13], develop sepsis [14], or are induced to differentiate into macrophages (Phorbol 12-Myristate 13-Acetate (PMA)) [15]. An interesting study conducted on human monocyte cell line THP-1 demonstrated that LPS, through the classical Toll-like receptor 4 (TRL4)- Myeloid Differentiation Primary Response 88 (Myd88)-dependent signaling pathway, induced increased HIF-1α transcription and protein stabilization in monocytes differentiated into macrophages. Interestingly, this activation was independent of NF-ĸB activity, but dependent on the presence of reactive oxygen species (ROS) [16]. The requirement for further stimulus (LPS) ensures that HIF-1α-driven inflammatory transcription occurs primarily in the context of infection or tissue injury, rather than systemic hypoxemia. Under normoxic conditions, but in an inflammatory context, ROS can stabilize HIF-1α by promoting its binding to the HRE element present in the *IL1B* promoter and inducing its transcription [17,18].

Another study on primary human monocytes showed that there is no stabilization/induction of HIF-1α, under either normoxic or hypoxic conditions, for up to 24 h, whereas in primary human monocyte-derived macrophages (MDMs), both HIF-1α and HIF-2α proteins are rapidly upregulated under acute hypoxic conditions (i.e., within 1 h of exposure) [19]. This study underlines that monocytes and macrophages may respond differentially to hypoxia and suggests that monocytes are likely to respond with molecular pathways that are not dependent on HIF-1α [19]. Instead, during monocyte–macrophage maturation, HIF-1α expression/activity increases, making macrophages more “HIF-1α-dependent” than monocytes [14,20,21]. In partial disagreement, another study demonstrated the induction of HIF1α transcription and its enhanced enzyme activity in primary human monocytes under acute hypoxic conditions (4 h) [22]. These authors showed an early alteration of metabolism (1 h), which could be the cause of HIF-1α upregulation, since metabolites such as lactate, succinate, and pyruvate promote HIF-1α stabilization through PHD inhibition [23,24]. However, the authors do not measure an increase in the pro-inflammatory cytokine TNF-α and IFN-α, nor an activation of NF-ĸB [22]. A recent study using single-cell reporter assays and reoxygenation kinetics indicated that the proteasome can rapidly change HIF-1α levels in cells exposed to acute hypoxia. Furthermore, the same study indicated a wide variability in HIF-1α levels within individual cells, with cells showing increased HIF levels and cells showing decreased levels [25]. All these studies suggest a certain degree of variability in individual responses to hypoxia (i.e., between different cells of a population and between different human subjects), which may depend on the pleiotropic nature of HIF signaling, the heterogeneity of monocyte responses, cellular metabolism, poor methodological standardization between studies, or, last but not least, the health status of the subject.

Prolonged exposure to hypoxia facilitates the differentiation of monocytes into macrophages, mainly of the M2-like anti-inflammatory phenotype. This may occur through increased lactate production, which promotes the activation of the enzyme matriptase involved in the cleavage/activation of protease-activated receptor 2 (PAR2), which is directly involved in monocyte–macrophage differentiation [26]. Subsequently, when monocytes are induced to differentiate into macrophages, the stabilization of the HIF-factors (HIF-1 and HIF-2) promotes the transcription of target genes, including the “ahif” transcript [27]. Ahif is a natural cis-antisense transcript of HIF-1α, which complements the 3′ untranslated end of the transcript, and in turn downregulates its half-life [28]. Therefore, under prolonged hypoxia, the abundant presence of the ahif transcript regulates the quantity of HIF-1α mRNA by promoting its degradation, and consequently, a drastic reduction in the protein, restoring homeostasis in the absence of tissue damage and any pathologies [27]. It is, therefore, clear that these responses can vary widely, from an adaptation to hypoxia, involving a molecular and metabolic reorganization aimed at restoring cellular homeostasis, to a hypoxic–inflammatory response that can lead to tissue damage and, ultimately, the development of pathologies. Unfortunately, the genetic and molecular bases characterizing these different responses are still unclear.

### 2.2. Early Signaling Events

**Figure 3:**
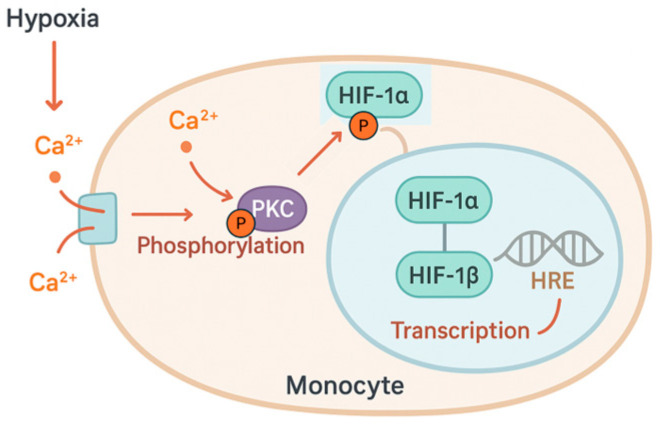
Hypoxia induces rapid increase in intracellular calcium (Ca^2+^), which activates protein kinase C (PKC). Activated PKC phosphorylates hypoxia-inducible factor 1α (HIF-1α), promoting its translocation to the nucleus. In the nucleus, the HIF complex enhances the transcription of target genes that respond to hypoxia.

Hypoxia causes a rapid increase in intracellular calcium. This increase results from both the extracellular compartment via calcium channels, and the release of calcium stored in the endoplasmic reticulum (ER), mediated by inositol-1,4,5-trisphosphate (IP_3_) receptors. Many kinases and phosphatases are calcium-dependent, including protein kinase C (PKC), which plays a central role in the early responses to hypoxia by the phosphorylation of target proteins (Figure 3).

**Figure 4:**
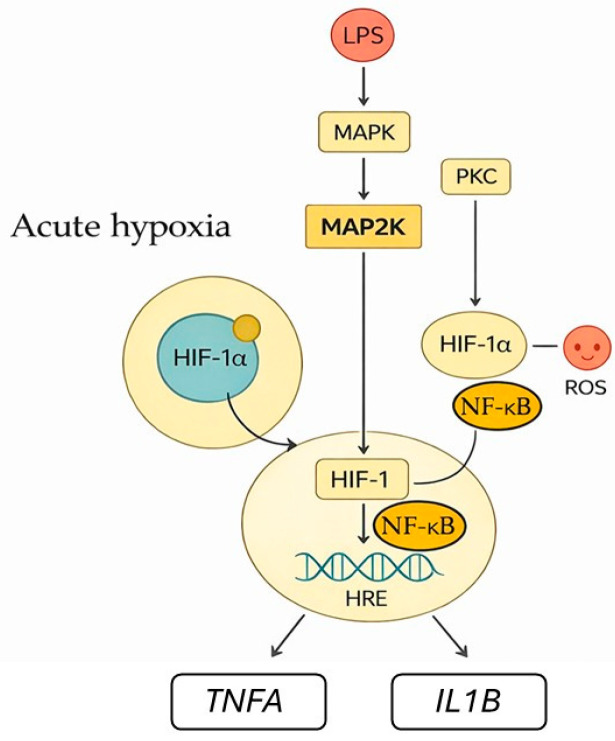
Acute hypoxia triggers signaling pathways that lead to HIF-1 activation and monocyte responses. PKC and MAPK/MAP2K pathways stimulated by inflammatory cues, like LPS, promote the stabilization and activation of cytoplasmic HIF-1α. Reactive oxygen species (ROS) enhance this response. PKC enhances NF-ĸB activation. Activated HIF-1α moves to the nucleus and forms a transcription factor complex with HIF-1β, regulating target genes expression such as *TNFA* and *IL1B*.

The PKC isoforms expressed in monocytes, PKC-α and PKC-β_1_, are particularly sensitive to acute hypoxia and contribute to cytoskeletal remodeling and HIF-1α activation [15,29]. One PKC substrate is focal adhesion kinase (FAK), which affects actin polymerization, adhesion, and cell motility. The Ca2^+^-dependent PKC signaling pathway promotes monocyte adhesion and migration, helping them reach hypoxic or inflamed tissues. Phosphorylation of cytoplasmic HIF-1α by PKC-α/β_1_ promotes its translocation to the nucleus by displacing cytosolic chaperones [30]. This mechanism modulates HIF transcriptional activity by making it dependent on the presence of important stress signals rather than oxygen levels alone. Furthermore, PKC interacts with other stress response pathways, including the mitogen-activated protein kinase (MAPK) and phosphoinositide 3-kinase (PI3K)/AKT cascades, aiming to amplify pro-survival and pro-inflammatory signaling (Figure 4).

PKC can activate NF-κB and increase cytokines expression, making monocytes more inflammatory under hypoxia. Fluctuations in intracellular calcium levels signal monocytes’ differentiation into macrophages, influencing the fate of monocytes under chronic hypoxia.

### 2.3. HIF-1α and NF-κB Crosstalk

Inflammation and hypoxia often occur simultaneously in damaged tissues, and they have many links in their signaling pathways [31,32,33]. Inflammation controls HIF activity, and hypoxia affects inflammatory response. The consequences of this relationship are not fully understood.

**Figure 5:**
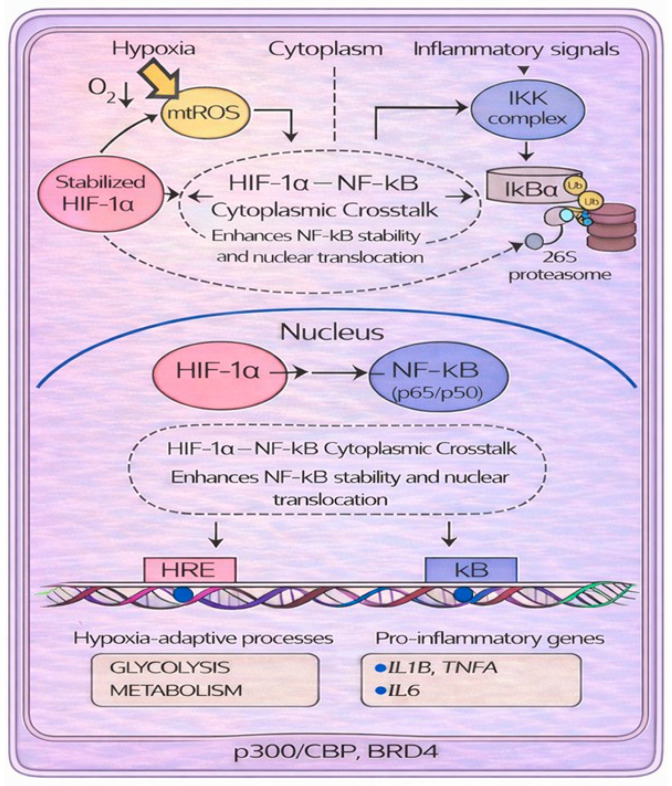
Molecular crosstalk between the transcription factors HIF-1α and NF-κB under hypoxic and inflammatory conditions. During hypoxia, the increase in reactive oxygen species (ROS) inhibits prolyl hydroxylase domains (PHDs), promoting the stabilization of HIF-1α. Reduced PHD activity also prevents hydroxylation-dependent inhibition of the IκB kinase (IKK) complex, allowing IKK to activate. Activated IKK phosphorylates IκBα, triggering its ubiquitination and proteasomal degradation; as a consequence, NF-κB moves to the nucleus. Stabilized HIF-1α interacts with NF-κB/p65 in the cytoplasm, enhancing NF-κB stability and nuclear translocation to induce genes involved in hypoxia adaptation (via HREs) and inflammatory responses (via κB elements).

NF-κB is the transcription factor that regulates inflammation. Under normal conditions, NF-κB in the cytoplasm is bound to the inhibitor of nuclear factor kappa B α (IκBα) [34]. In response to inflammatory stimuli and infections, an IκB kinase (IKK) phosphorylates IκBα, promoting its degradation by the proteasome through ubiquitination [35]. When IκBα is degraded, NF-κB moves to the nucleus and binds to consensus sequences (κB) within the promoter of target genes [36,37]. During acute hypoxia, NF-κB is activated early, through mitochondrial ROS and IKK-dependent degradation of IκBα. NF-κB can recognize the consensus sequence in the *HIF1A* gene and induces HIF-1α mRNA expression [38]. When HIF-1α and NF-κB are together in the cytoplasm, they interact (Figure 5).

NF-κB moves to the nucleus and increases its transcriptional activity [39]. Furthermore, they may share transcriptional coactivators, such as p300/CBP and Bromodomain Containing 4 (BRD4), required for the induction of genes involved in both hypoxia (through HREs) and inflammation responses (through κB element) [33]. Not least, the HIF and NF-κB signaling pathways share some regulators. For example, PHD targets IKK, which regulates NF-κB activity. Under normoxia, PHD hydroxylates IKKβ, promoting its ubiquitination and degradation. Accordingly, IĸB remains bound to NF-κB, preventing its translocation to the nucleus. However, when oxygen is depleted (hypoxia), PHD is inhibited and IKK escapes degradation. The IKK complex removes the inhibitory IκB from NF-κB, promoting its translocation to the nucleus and inflammatory gene expression [40,41]. Acute hypoxia also quickly activates NF-κB in monocytes, linking oxygen sensing to pro-inflammatory responses. Several mechanisms contribute to this rapid activation [31]. Another point of connection between the two signaling pathways is represented by FIH, which can hydroxylate IκBα. However, this modification does not appear to have regulatory direct effects on NF-κB. Conversely, IκBα promotes HIF-1α activity in two ways: (1) it inhibits FIH-mediated hydroxylation, and (2) it sequesters FIH under hypoxic conditions, preventing it from binding to HIF-1α and thus allowing its translocation to the nucleus [42]. During hypoxia, mitochondria generate ROS (mtROS). These ROS activate IKK, which phosphorylates, ubiquitinates, and degrades IκBα, allowing NF-κB (p65/p50) to enter the nucleus [43,44]. Furthermore, mtROS contribute to HIF-1α stabilization via two distinct mechanisms: first, by enabling the nonenzymatic decarboxylation of the PHD substrate [43], and second, by oxidizing the PHD cofactors Fe2^+^ to Fe3^+^ [44]. Finally, ROS inhibit FIH-mediated hydroxylation at a site distinct from the one targeted by PHDs. This inhibition compromises HIF-1α function by reducing the activity of its C-terminal transactivation domain (CAD) [45]. The two different sites at which HIF-1α can be hydroxylated also differ in sensitivity to hydrogen peroxide concentrations and oxygen tension, suggesting that hypoxia and ROS may provide different levels of regulation of HIF-1α stability [45].

The bidirectional NF-κB-HIF-1α interplay also creates potential points of pathological amplification (for example in sepsis or in chronic inflammatory diseases), making components of these early signaling pathways attractive targets for therapeutic modulation.

### 2.4. Cytokines and Chemokines Induction

**Figure 6:**
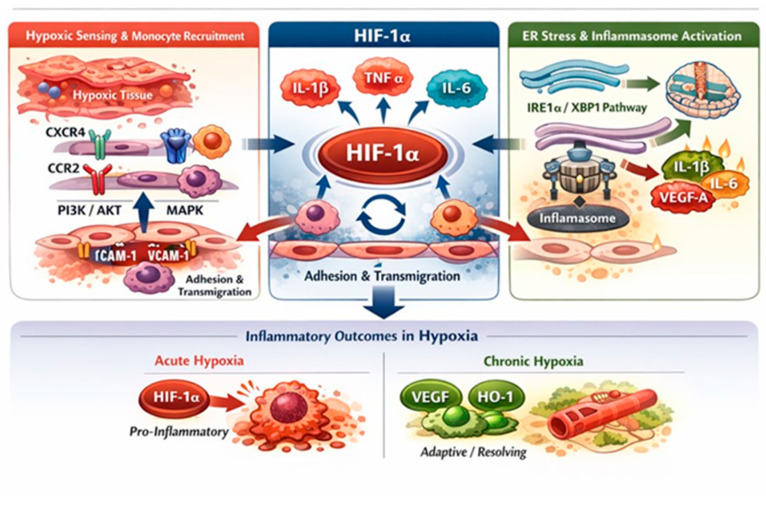
Hypoxia-driven regulation of monocyte migration and inflammatory responses. Hypoxia promotes monocyte recruitment by upregulating chemokine receptors (CXCR4, CCR2) and activating PI3K/AKT and MAPK signaling, thereby enhancing migration and survival. Concurrently, hypoxia-induced endothelial activation increases ICAM-1 and VCAM-1 expression, facilitating monocyte adhesion and trans-endothelial migration. Hypoxia stabilizes HIF-1α, a central regulator of inflammatory responses that induces pro-inflammatory cytokines, including IL-1β, TNF-α, and IL-6, directly or through crosstalk with NF-κB. Endoplasmic reticulum stress further amplifies HIF-1α-dependent cytokine production via the IRE1α/XBP1 pathway and inflammasome activation. The inflammatory outcome is context-dependent, with acute hypoxia favoring pro-inflammatory responses and chronic hypoxia promoting adaptive and resolving programs characterized by VEGFA and heme oxygenase-1 (HO-1) protein expression.

Monocytes migrate to hypoxic tissues, because they overexpress chemokine receptors like CXCR4 [46]. This receptor is key to cell migration, proliferation, and inflammation [47,48]. A compelling study involving 11 volunteers compared the response of purified primary human monocytes exposed to in vitro hypoxia with the effects of 24 h in vivo hypobaric hypoxia in the same subjects. In vitro exposure led to an upregulation of IL-1β and C-C chemokine receptor 2 (CCR2). Similarly, in vivo experiments demonstrated a significant increase in IL-1β, CCR2, and CXCR4 expression [49]. The signaling pathways downstream CXCR4, i.e., PI3K/AKT [50] and MAPK [51], influence migration, cell proliferation, and survival. In ischemic tissues, endothelial cells play an important role in facilitating monocyte trans-endothelial migration and monocyte adhesion, since they can increase the expression of intercellular adhesion molecule-1 (ICAM-1) and vascular adhesion molecule-1 (VCAM-1) [52] (Figure 6).

Monocytes exposed to hypoxia upregulate adhesion molecules (like CD11a, CD11b, CD11c, CX3CR1) [53]. Consequently, a key pathway triggered in the early stages of hypoxia is responsible for regulating monocyte recruitment. This is not unexpected, as circulating monocytes rapidly sense the lack of oxygen and exploit the enhanced cytoskeletal remodeling and chemotactic responsiveness to exit the bloodstream, migrate to tissues where they can differentiate into effector macrophages, and initiate the early stages of the adaptive response.

Regarding cytokines, HIF-1α induces the transcription of key pro-inflammatory cytokines, including IL-1β, TNF-α, and IL-6, through direct binding to the HRE in the promoters, or via crosstalk with NF-κB [17,54]. HIF-1α and IL-1β are functionally linked, allowing for mutual regulation of their signaling pathways. In mouse bone marrow-derived macrophages (BMDMs) exposed to hypoxia (24 h), LPS-induced succinate stabilized HIF-1α and stimulated IL-1β protein expression, while TNF-α remained unchanged, and IL-6 expression decreased [54]. Inhibition of HIF-1α significantly reduces LPS-mediated IL-1β production, suggesting that HIF-1α regulates the inflammatory response through an increase in IL-1β. On the other hand, IL-1β and TNF-α can promote HIF-1α transcription and stabilization even under normal oxygen levels [39,54,55].

Another interesting interaction is that between endoplasmic reticulum (ER) stress and HIF-1α (Figure 6). ER stress-associated pathways may synergize with HIF-1α to adapt to oxygen starvation. Activation of the ER stress pathway (IRE1α/XBP1) in immune cells promotes HIF1α-dependent cytokines production, including IL-1β, IL-6, and VEGF-A [56]. Increased IL-1β production activates inflammasome responsible for its maturation. This sequence links hypoxic signaling to inflammasome activation, amplifying IL-1β maturation and secretion. The inflammasome is considered the central hub linking innate immune sensitivity to systemic inflammation, and now represents a cutting-edge therapeutic target. Similarly, HIF-1α also contributes to sustained inflammation through TNF-α and IL-6, leading to endothelial activation and monocyte recruitment in hypoxic tissues [52]. Blood monocytes and monocyte-derived macrophages (MDMs) from patients with highly active sarcoidosis, exposed in vitro to hypoxia for 24 h, show a pro-inflammatory response, including TNF-α and HIF-1α-dependent IL-1β production without NF-ĸB activation [57]. A recent study found that at high altitudes, human subjects produced more pro-inflammatory cytokines like TNF-α, IL-17A, IL-2, IL-6, IFN-γ, and fewer anti-inflammatory cytokines like IL-10. Interestingly, TNF-α levels increased at low altitudes and predicted acute mountain sickness (AMS). At middle high-altitude (3700 m), IL-2 and IL-17 cytokines predicted AMS [58]. A study of the Gene Expression Omnibus (GEO) dataset found that monocytes, M1-type macrophages, and NK cells drove inflammatory response in AMS. Specifically, the genes *IL15RA*, *CD5*, *B-cell Activating Factor* (*BAF*), *IL21R*, *JAK2*, and *CXCR3* were identified as central hub genes within the bioinformatic network. Notably, JAK2 was positively correlated with monocytes and played a key role in regulating macrophage polarization and homeostasis [59]. Single-cell profiling of patients with high-altitude pulmonary hypertension (HAPH) revealed increased non-classical and intermediate monocyte subtypes with high levels of pro-inflammatory cytokines production like IL-2, IL-4, and IL-13. Notably, these subsets displayed broad functional impairments such as phagocytosis, together with a marked downregulation of HIF-1α, suggesting a maladaptive immunometabolic response to chronic hypoxia [9].

However, HIF-1α can have anti-inflammatory effects under certain conditions [60]. For example, under chronic hypoxic conditions, prolonged HIF-1α activity can induce the expression of VEGF-A and heme oxygenase-1 (HO-1) proteins, promoting tissue remodeling and resolution [61,62] (Figure 6). HIF-1α favors pro-inflammatory phenotype, while HIF-2α supports anti-inflammatory functions [10,63]. Ultimately, the production of pro- and anti-inflammatory cytokines in hypoxia depends on multiple factors, some of which are known, while others are still obscure.

### 2.5. Epigenetic Regulation

The regulation of gene expression during hypoxia may depend on the activity of histone-modifying enzymes, as these enzymes can methylate/demethylate not only histone proteins, but also non-histone proteins. Modification of transcription factors can affect their stability, and this is what happens to HIF-1α under hypoxic conditions. Histones-modifying enzymes belong to a large family including histone demethylases (KDMs), like JmjC domain-containing histone demethylases (JMJD1A, JMJD2B, KDM3A, and KDM4), and histone deacetylases (HDACs) [64]. Demethylases and deacetylases play key roles in regulating gene expression associated with angiogenesis, anaerobic glycolysis, and epithelial–mesenchymal transition (EMT), all biological processes that are part of the response to hypoxia. For example, LSD1 demethylates HIF-1α at K32 and K391 residues in response to hypoxia-mimicking conditions and facilitates its stabilization by acting on VHL-induced HIF-1α degradation [65]. Under hypoxic conditions, some JMJC demethylases are inhibited in their enzymatic activity because they require oxygen to remove methyl groups and make chromatin accessible, but, interestingly, their expression increases, perhaps to compensate for the reduced enzymatic activity [66]. HIF-1α upregulates the expression of the demethylases KDM3A and KDM4B in monocyte–macrophages. However, their enzymatic activity remains inhibited, as evidenced by increased methylation in the promoter regions of target genes like *CCL2*, *CCR1*, and *CCR5*, which play key roles in monocyte recruitment and migration [67]. However, not all demethylases are inactivated under hypoxic conditions. Some KDMs retain their enzymatic activity and can promote the expression of target genes by stabilizing HIF or removing repressive marks, such as H3K9me3, from promoter regions containing HREs [64]. Gene-specific changes due to histone modifications depend on multiple factors, such as cell type and duration of hypoxia. Chronic hypoxia and repeated episodes of hypoxia induce a different response in immune cells than acute hypoxia. Monocytes repeatedly exposed to hypoxia, for example, can undergo epigenetic reprogramming, altering their response to subsequent stimuli, a phenomenon known as hypoxia-induced trained immunity [68,69,70].

## 3. Conclusions

Acute hypoxia initially promotes a pro-inflammatory phenotype, and its effects are transient. Indeed, as soon as oxygen levels return to normal, HIF-1α is rapidly degraded by PHD enzymes, leading to the restoration of cellular homeostasis, due to the reduction in the expression of glycolytic genes and cytokines. Furthermore, monocytes can produce anti-inflammatory mediators, like the cytokine IL-10 and adenosine, preventing excessive tissue damage [71].

At the molecular level, acute hypoxia triggers a coordinated and sequential activation of interconnected signaling pathways. Early hypoxic stress induces a rapid increase in intracellular Ca^2+^ levels, which represents one of the first signaling events and leads to the activation of calcium-dependent kinases, particularly PKCα and PKCβ_1_. PKC activation functions as an upstream signal linking oxygen deprivation to cytoskeletal remodeling, cell migration, and transcriptional regulation. Through phosphorylation of cytoplasmic HIF-1α, PKC facilitates its dissociation from cytosolic chaperones and promotes its nuclear translocation, thus coupling HIF-1α activation to stress signaling rather than oxygen availability alone.

Hypoxia-induced mitochondrial ROS production acts as a critical amplifier of both HIF-1α and NF-κB signaling. ROS-mediated inhibition of PHD enzymes stabilizes HIF-1α, while reduced PHD-dependent hydroxylation of the IKK complex allows for NF-κB activation through IκBα degradation. PKC further enhances NF-κB signaling, establishing a functional convergence between PKC and NF-κB pathways under hypoxic conditions. NF-κB activation promotes the transcription of pro-inflammatory cytokines and chemokines, including IL-1β, TNF-α, CCR2, and CXCR4, thereby facilitating monocyte recruitment, survival, and inflammatory amplification. Once stabilized, HIF-1α and NF-κB engage in a bidirectional crosstalk that reinforces hypoxia-driven inflammation. NF-κB induces HIF-1α transcription, while HIF-1α cooperates with NF-κB at both cytoplasmic and nuclear levels, sharing transcriptional coactivators such as p300/CBP and BRD4. This coordinated interaction ensures that cytokine and chemokine production is tightly coupled with metabolic reprogramming and hypoxic adaptation, integrating inflammatory signaling with cellular survival programs.

When acute hypoxia hits a specific tissue like the myocardium (infarction), the brain (stroke), or the lung (pulmonary embolism and acute respiratory distress syndrome (ARDS)), monocytes rush in, differentiate into macrophages, and contribute to both inflammation and tissue repair [20,71,72]. In these contexts, HIF-1α contributes to inflammation by promoting cytokine production, and prolonged and sustained activation can degenerate into chronic inflammation that, if not resolved, becomes pathological. Researchers aim to modulate HIF-1α activity in monocytes, either with PHD inhibitors or via metabolic control [73,74].

In general, primary blood monocytes exposed to hypoxia are less studied than macrophages. In particular, the hypoxic conditions required for functional HIF-1α activation in circulating monocytes are unclear: threshold, duration, and intensity of stimulus. In contrast, macrophages have well-established transcriptional and post-transcriptional programs triggered by hypoxia through HIF-1α activity [21,75]. Transcriptomic analyses suggest distinct gene expression profiles between monocytes and macrophages, highlighting unique and transient hypoxia response programs in monocytes [76]. In conclusion, in monocytes exposed to acute hypoxia, HIF-1α plays a dual role: adapting to hypoxia and the pro- and anti-inflammatory response. Future research should investigate the factors and cofactors that determine HIF activation in monocytes and the outcome of hypoxia. This knowledge will allow us to develop intervention strategies to limit and/or reverse hypoxia damage.

## Data Availability

No new data were created or analyzed in this study. Data sharing is not applicable to this article.
